# S1-5 Pupil’s experiences of affordances in school-based physical programmes in Norway and Estonia

**DOI:** 10.1093/eurpub/ckad133.008

**Published:** 2023-09-11

**Authors:** Eirini Pardali

**Affiliations:** University of Southern Norway

## Abstract

**Purpose:**

This study aimed to examine pupils’ experiences of the school environment and how it stimulated different physical activity behaviours at selected Norwegian and Estonian schools involved in school-based physical activity programmes.

**Methods:**

Pupils from elementary schools (10-year-olds, n = 21) and lower-secondary schools (15-year-olds, n = 17) provided 66 photos of their school environment and participated in 9 focus group interviews. The data were reflexively thematically analysed using Gibson’s theory of affordances

**Results:**

The findings indicate that the pupils’ physical activity behaviour was promoted and constrained by two primary factors: physical affordances of the active components (indoor/outdoor affordances, seasonal affordances) and social affordances (school rules, imaginative play, togetherness, the relationship between pupils and teachers, pupils’ choice). The 10-year-old pupils in both countries experienced their physical activity behaviour to be promoted and constrained predominantly by physical affordances, their relationship with their teachers, rules and imaginative play. For the 15-year-old pupils, the primary promoting and constraining factors were physical affordances, their relationship with their teachers, togetherness and having choice during physically active learning. Thus, the pupils considered the design of the physical and social environment within the school-based physical activity programmes to influence their physical activity behaviour.

**Conclusions:**

It is suggested that school environments with diverse, indoor/outdoor, spacious, multifunctional and natural spaces that facilitate physical activity alongside socialisation can motivate pupils to be physically active. In addition to an emphasis on physical affordances, a focus on open, trusting and engaging relationships between teachers and pupils and the consideration of pupils’ voices within school-based physical activity programmes can promote physical activity behaviour and consequently programme sustainability.

**Funding:**

This study was partially funded by the Increasing Physical Activity of Schoolchildren project, financed by the European Economic Area (EEA) Grants (grant 2014-2021.1.05.20-0004) under the Local Development and Poverty Reduction programme, co-financed by the Ministry of Social Affairs and self-financed by the University of Tartu. Additionally, the first author received funding for a PhD fellowship from the University of South-Eastern Norway.

